# Local allergic rhinitis^☆^

**DOI:** 10.1016/j.bjorl.2016.09.001

**Published:** 2016-09-12

**Authors:** João Ferreira de Mello Junior

**Affiliations:** Universidade de São Paulo (USP), Faculdade de Medicina, São Paulo, SP, Brazil

Allergic rhinitis (AR), by definition, denotes an inflammation of the nasal mucosa, mediated by IgE, accompanied by symptoms such as nasal congestion, local itching, sneezing and rhinorrhea.^1^

There have been two large studies assessing the prevalence of AR in Brazil. The first, through questionnaires applied to children and adolescents, revealed that nasal and ocular symptoms during the previous year, but without the presence of an upper respiratory infection were present in approximately 12–15% of subjects. The second, also using questionnaires, but evaluating all age ranges with a diagnosis of AR, found a prevalence of 9%.^2^, ^3^

Interestingly, these studies infer that the nasal symptoms are allergic in nature, since the presence of the specific IgE has not been proven, a basic assumption to determine an allergic etiology (see definition).

In 1859, Charles Harrison Blackley applied pollen to his nasal mucosa and that triggered the symptoms of rhinitis. Thus, the nasal challenge test for the diagnosis of allergic rhinitis appeared.^4^ Other methods are also used to study this class of antibodies such as the skin prick testing and specific serum measurements (immunoenzymatic and immunofluorometric assays).^2^

One of the first studies to demonstrate the production of specific IgE in nasal secretion only was published in 1975. Fourteen patients with clinical symptoms suggestive of AR caused by dust mites, although with a negative radioallergosorbent test (RAST) were submitted to nasal challenge testing with this antigen. All patients had nasal symptoms, which were absent in the control group.^5^

This fact demonstrates that there are two phenotypes of AR. One represents “classic” patients, who exhibit a characteristic clinical picture, the presence of positive systemic specific IgE in skin and serum tests, and have a personal and family history of atopy. The other phenotype contains individuals who have symptoms only after contact with allergens, but no family history of atopy and positive nasal provocation test only for the antigen. These individuals have what is called Local Allergic Rhinitis (LAR).^6^

The prevalence of LAR is not known. Some studies show that over 47% of cases of noninfectious, non-allergic rhinitis may be a result of specific IgE production restricted to the nasal mucosa.^7^ Being an unknown phenotype, it is underdiagnosed so one can potentially assume that the prevalence of an allergic etiology in nasal symptoms is much higher than what is currently proven.

There is very little in-depth medical literature on LAR; however, the questions of whether LAR represents a possible early onset of AR has been addressed. Follow-up of adult and adolescent patients with LAR for 5 years did not show any difference in the onset of atopy in (6.25%) when compared to controls (5.2%).^8^

Patients with LAR have the same classic symptoms of those with AR, such as sneezing, itching, nasal obstruction and rhinorrhea.^8^ A study comparing patients with AR and LAR also showed that both share a similar clinical and demographic phenotype. They occur preferentially in nonsmokers, female individuals who exhibit a severe persistent clinical picture, often with conjunctival and asthma symptoms;the house dust mite (*Dermatophagoides pteronyssinus*) is the main associated agent.^9^

In addition to the dust mites, other antigens are associated with LAR, such as pollens and fungi, causing symptoms in both children and adults.^10^, ^11^

From a pathophysiological point of view, LAR is similar to AR. An inflammatory infiltrate pattern with Th2 (IgE-mediated clinical pictures), immediate phase characterized by the activation and release of basophil mediators and a late phase with eosinophil attraction and activation can be observed.^10^

Regarding therapy, LAR patients respond well to both oral and topical medications. Additionally, they also show improvement with allergen-specific subcutaneous immunotherapy, similarly to patients with AR. A pilot study with 20 patients with LAR sensitive to grasses were submitted to allergen-specific subcutaneous immunotherapy. At the end of the study, the authors observed greater tolerance to the allergen and symptom reduction, as well as decreased use of rescue medication.^12^ Currently, double-blind placebo-controlled studies are being performed with grass and house dust mite antigens.^7^

The diagnosis of LAR is corroborated by demonstrating the presence of the nasal specific IgE through nasal provocation test with allergens or the local synthesis of IgE, without the presence of systemic atopy.^7^ The nasal secretion lavage is very useful to assess cellularity, presence of inflammatory mediators and specific IgE.^7^ The nasal challenge testing with allergens reproduces the “natural” allergic reaction, demonstrating the immediate and late phases, the sensitization to the allergen and its importance in symptom onset.^7^ In clinical practice, it is considered the gold standard for the diagnosis of both AR and LAR; however, it is not a routinely performed test, as it is time-consuming and requires well-trained staff.^7^ Therefore, the rapid development of an inexpensive method for the determination of specific IgE in nasal secretion is important, so we can learn the true prevalence of this phenotype and establish the correct and appropriate diagnosis and treatment in our patients.

## Conflicts of interest

The authors declare no conflicts of interest.
